# Long-Term Follow Up of the Erectile Function of Testicular Cancer Survivors

**DOI:** 10.3389/fendo.2019.00196

**Published:** 2019-04-02

**Authors:** Francesco Pallotti, Alessandra Petrozzi, Francesco Cargnelutti, Antonio Francesco Radicioni, Andrea Lenzi, Donatella Paoli, Francesco Lombardo

**Affiliations:** ^1^Laboratory of Seminology–Sperm Bank Loredana Gandini, Department of Experimental Medicine, Sapienza University of Rome, Rome, Italy; ^2^Hormone Laboratory, Department of Experimental Medicine, Sapienza University of Rome, Rome, Italy

**Keywords:** testicular cancer, cancer survivors, orchiectomy, sexual function, erectile dysfunction, IIEF

## Abstract

The diagnosis of testicular cancer (TC) can have a considerable and persistent impact on a patient's sexuality, especially given its location. The high prevalence of TC in young adults, and the good prognosis, explain the great interest in sexual dysfunction and its influence on post-treatment quality of life. The aim of this study was to evaluate the impact of the diagnosis and treatments (inguinal orchiectomy and chemotherapy) on sex life. For this purpose, we recruited 241 TC patients attending the Laboratory of Seminology–Sperm Bank “Loredana Gandini” for sperm cryopreservation (mean age 31.3 ± 6.9 years) and 223 cancer-free healthy men who were undergoing andrological screening (mean age 32.0 ± 7.7 years). The IIEF-15 questionnaire was administered at the baseline (post-orchiectomy, pre-chemotherapy—T0) and at 6 (T1), 12 (T2), 18 (T3), 24 (T4), 48 months (T5) and >5 years (T6, median 96 months) after chemotherapy to all patients, to evaluate the following domains: erectile function (EF), orgasmic function (OF), sexual desire (SD), intercourse satisfaction (IS) and overall satisfaction (OS). A subgroup of patients also underwent blood sex hormone analysis for further correlations with IIEF scores. At the baseline, 37.7% of patients had erectile dysfunction (EF score <26) and all IIEF domains except OF showed significantly lower scores than in controls (*p* < 0.001). Long-term follow-up revealed persistently lower scores in TC survivors than in controls for EF, SD, IS, and OS. Furthermore, most IIEF domains did not improve significantly in TC patients during the duration of the follow-up, with the exception of EF, which showed a significant improvement from T2. Finally, no significant correlation was found between hormone levels (gonadotropin and testosterone) and IIEF-15 scores. In conclusion, TC and its treatment have a significant effect on sexuality. The absence of a clear correlation with biochemical hypogonadism suggests that this may to a large extent be due to the surgical procedure itself, or to the psychological impact of a cancer diagnosis.

## Introduction

Alongside cardiovascular disease, cancer is currently the main cause of mortality worldwide. Italian cancer registers show that nearly 5% of the population has received a diagnosis of cancer (1). However, modern treatments mean that the life expectancy of about 60% of juvenile and young adult cancer survivors is comparable to that of the general population.

Men in reproductive age are mainly affected by testicular cancer (TC) and lymphomas, but despite the high incidence, their 5-year survival rates are above 80–90% (2, 3). These cancer survivors will therefore have to live with the long-term physical and psychological consequences of both their treatments (surgery, chemotherapy, radiotherapy) and the diagnosis itself (4, 5). This has important health, social and economic repercussions, as these long-term consequences affect men in their working and reproductive years, affecting their physical capabilities as well as their reproductive and sexual health. According to the WHO, reproductive health is defined as a “state of complete physical, mental and social well-being and not merely the absence of disease or infirmity, in all matters relating to the reproductive system, and to its functions and processes” (6). Sexual health should in fact be considered as a complex interaction of multiple factors including social and cultural aspects, individual experiences, and self-image. Cancer and its treatments should certainly be considered as capable of disrupting sex life, but many patients find it difficult to discuss these problems and there is a lack of consensus on valid outcome measures for assessing sexual function in cancer patients on the basis of a broader definition of sexual health (7)—issues yet to be faced in common practice or research.

Most knowledge of male sexual dysfunction after cancer pertains to prostate cancer after invasive surgical procedures and hormonal treatments: however, this is not representative of other situations (8). The sexological features of testicular cancer have been investigated by several authors, revealing associations with perceived loss of masculinity and sexual function, which paves the way for psycho-organic sexual dysfunctions (4). Orchiectomy itself can alter the perception of body image, potentially manifesting as reduced libido and sexual gratification linked to the psychological stress of not “being normal” (9). The cancer diagnosis itself is a moment of intense psychological distress and, although literature reports vary widely, about one-third of patients with testicular cancer report erectile dysfunction and/or ejaculation disorders (10). Invasive and destructive surgery such as retroperitoneal lymph node dissection increases the frequency of such dysfunctions (10–12). However, most data focus on either the short- or the long-term consequences of therapy and reports of thorough longitudinal follow-up from diagnosis to long-term survivorship are rare. The aim of this study is to evaluate the effect of TC after orchiectomy and provide a complete follow-up in order to highlight possible short- and long-term sexological changes after treatment.

## Materials and Methods

### Patients

The study was approved by our University Hospital's institutional review board (Ethical Committee Policlinico Umberto I—University of Rome “Sapienza”) and all patients gave informed written consent. We recruited 241 sexually active consecutive patients (mean age 31.3 ± 6.9 years, range 18–52) with a recent diagnosis of testicular cancer who attended the Laboratory of Seminology—Sperm Bank “Loredana Gandini” between 2006 and 2018 for sperm cryopreservation before any cancer treatment. All patients had undergone orchiectomy within the previous 30 days.

As the control group, we recruited 223 healthy subjects (mean age 32.0 ± 7.7 years, range 18–55) who attended the Endocrinology and Andrology outpatient clinic of the Department of Experimental Medicine in the same period for idiopathic primary infertility. Subjects with hypogonadism and other endocrine disorders, diabetes, hypertension, cryptorchidism, history of cancer and/or previous chemo/radiotherapy, history of urogenital surgery, Klinefelter Syndrome and other chromosomal abnormalities or any genetic diseases were excluded. Both patients and controls underwent a thorough medical history and a general and andrological physical examination, and were administered the International Index of Erectile Function 15 questionnaire (IIEF-15) to evaluate sexual function. The IIEF-15 was administered to TC patients at the post-orchiectomy baseline before chemotherapy (T0) and at 6 (T1), 12 (T2), 18 (T3), 24 (T4), and 48 months (T5) after chemotherapy, with a final follow-up between 5 and 12 years post-chemotherapy (T6, median 96 months). Each patient underwent the baseline evaluation and at least one follow-up. A subgroup of TC patients also underwent blood hormone tests (FSH, LH, total Testosterone) for later comparison with healthy controls to investigate any correlations with IIEF scores. This subgroup patients underwent blood hormone analysis at T0, T1, and T2.

### Hormone Analysis

Blood samples were collected at 8.00 a.m. after at least 8 h of overnight fasting for measurement of FSH, luteinizing hormone (LH) and total testosterone. Serum FSH, LH, and testosterone were measured by chemiluminescent microparticle immunoassay (CMIA, Architect System; Abbott Laboratories, Abbott Park, IL, USA), with detection limits of 0.05 mIU/ml, 0.07 mIU/ml, and 0.28 nmol/l, respectively. Intra- and inter-assay coefficients of variation were 3.1 and 7.0% at 3.2 mIU/ml (FSH), 3.6 and 5.1% at 3.3 mIU/ml (LH), and 2.1 and 3.6% at 10.08 nmol (total testosterone). Normal ranges for adults were 1.38–9.58 mIU/ml (FSH), 1.80–8.16 mIU/ml (LH), and 9.4–33.5 nmol/l (total testosterone).

### IIEF-15

Sexual function can be evaluated in a reassuring and comfortable setting with self-administered questionnaires. One of the most widely used in both clinical practice and research is IIEF-15. This multidimensional tool enables the rapid, reliable and reproducible measurement of several domains of sexual function (13). It was developed to enable the evaluation of patients' sexuality in clinical trials for erectile dysfunction with high sensitivity and specificity. The advantage of self-administration is that it is perceived by patients as less invasive and burdensome than a direct interview. The classic form has 15 items grouped into five domains: erectile function (EF), questions 1–5 and 15; orgasmic function (OF), questions 9–10; sexual desire (SD), questions 11–12; intercourse satisfaction (IS), questions 6–8; and general satisfaction (GS), questions 13–14. Generally, a score below 26 in the EF domain is considered diagnostic for erectile dysfunction.

### Statistical Analysis

Continuous variables are presented as mean, median and standard deviation. Differences between groups were evaluated by ANOVA or Kruskal-Wallis test, based on data distribution as evaluated by Kolmogorov-Smirnov test. *Post-hoc* results were corrected using the Bonferroni method for multiple comparisons. Categorical variables are presented as counts and percentages and were compared by χ^2^ test. Statistically significant correlations among the variables examined were evaluated using Spearman's rank correlation test. The probability values are 2-sided and a *p*-value < 0.05 was considered statistically significant. All computations were carried out with Statistical Package for the Social Sciences (SPSS) 25.0 (SPSS Inc., Chicago, USA).

## Results

### Pre-therapy

Table 1 describes the demographics of the recruited TC patients and control subjects. The TC and control groups were comparable in age, BMI and percentage of smokers. The baseline prevalence of erectile dysfunction as self-reported through the IIEF (EF domain score < 26) was 37.8% (91/241) in TC patients against 9.9% (22/223) in the control group (χ^2^ *p* < 0.001). Erectile dysfunction was severe in 23.2% (56/241), moderate in 4.1% (10/241) and mild in 10.4% (25/241) of TC patients, while all cases in the control group were mild. The baseline comparison of TC and CTR groups is presented in Table 2: all IIEF-15 domain scores were significantly worse in patients than in controls (all *p* < 0.001), with the exception of orgasmic function (*p* = 0.334). No significant correlations were found between IIEF scores and age, BMI, smoking status, cigarettes smoked/day and years of smoking in either group.

**Table 1:** Testicular cancer and Control group demographics: continuous data are presented as mean ± SD, median (in brackets) and 25–75th percentile of data distribution; categorical data as percentage and count.

	**Testicular cancer (241 pts)**	**Controls (223 pts)**
Age at diagnosis (years)	31.3 ± 6.9 (31.0)	32.0 ± 7.7 (32.0)
	26.0–36.0	26.0–37.0
BMI (kg/m^2^)	24.9 ± 3.0 (24.5)	24.6 ± 2.7 (24.1)
	23.0–26.7	22.7–25.9
Smokers	19.5%	23.3%
	47 pts	52 pts
Cigarettes/day^a^	11.4 ± 8.5 (10.0)	11.3 ± 8.5 (10.0)
	5.0–15.0	5.0–15.0
Years of smoking^a^	12.3 ± 6.6 (10.0)	10.6 ± 6.2 (10.0)
	7.0–16.0	6.0–15.0
Occupation	Office worker (23.6%) Factory/heavy worker (15.3%) Freelance professional (13.9%) Student/university (9.7%) Police/military (2.8%) Driver (5.6%) Healthcare professional (4.2%) Unemployed (5.6%) Other (19.3%)	Office worker (18.1%) Factory/heavy worker (16.9%) Freelance professional (20.5%) Students/university (14.5%) Police/military (3.6%) Driver (3.6%) Healthcare professional (9.6%) Unemployed (2.4%) Other (10.8%)
Histological diagnosis	58.5% Seminoma pT1-pT2 30.7% Mixed germ cell tumor pT1-pT2 8.0% Embryonal carcinoma pT1-pT2 2.8% Yolk sac tumor	/
Chemotherapy regimen	BEP 1-3 cycles cysplatin 1 cycle	/

a *smokers only*.

**Table 2:** Baseline testicular cancer IIEF scores vs. control group: continuous data are presented as mean ± SD, median (in brackets) and 25–75th percentile of data distribution.

	**ED domain**	**OF domain**	**SD domain**	**IS domain**	**GS domain**
TC	22.7 ± 9.1 (27.0)	8.2 ± 1.9 (10.0)	7.5 ± 1.9 (8.0)	8.3 ± 4.7 (10.0)	7.4 ± 2.6 (8.0)
241 pts	20.0–29.0	8.0–10.0	6.0–9.0	7.0–12.0	6.0–10.0
CTR	27.9 ± 2.6 (28.5)	8.9 ± 1.2 (10.0)	8.9 ± 1.2 (9.0)	12.6 ± 1.9 (13.0)	9.0 ± 1.3 (9.0)
223 pts	27.0–30.0	8.0–10.0	8.0–10.0	11.5–14.0	8.0–10.0
*P*-value	< 0.001	0.334	< 0.001	< 0.001	< 0.001

*(Mann Whitney U test). EF, erectile function; OF, orgasmic function; SD, sexual desire; IS, intercourse satisfaction; GS, general satisfaction*.

### Post-therapy

**Figure 1:**
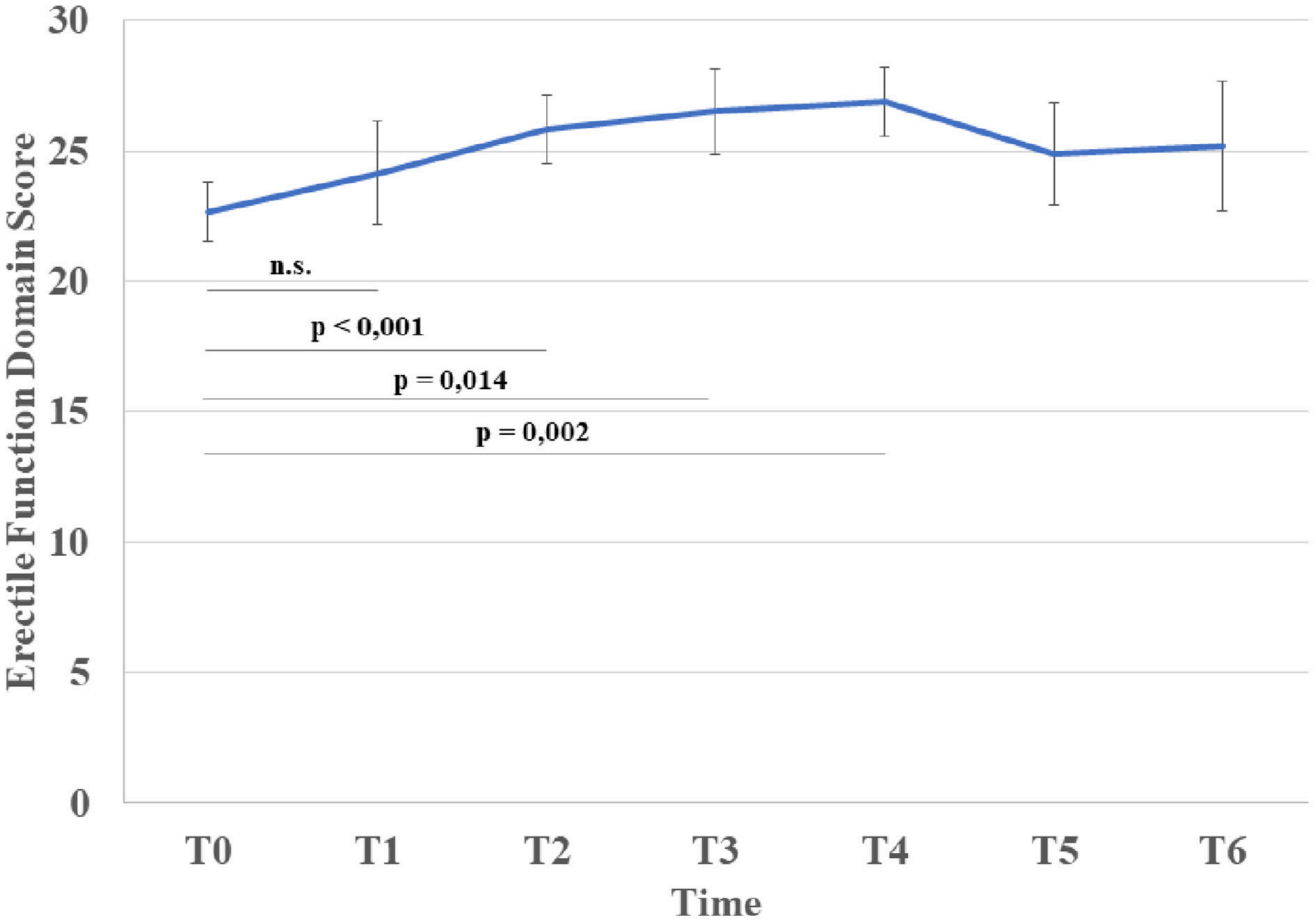
Variation in mean erectile function domain score and statistical significance from T0 (Bonferroni correction for multiple comparisons).

All patients underwent a chemotherapy regimen only, as indicated in Table 1. IIEF scores from longitudinal follow up are compared against healthy controls are shown in Table 3. Kruskal Wallis test with *post-hoc* corrections for multiple comparisons (Bonferroni) revealed that:

- ED domain scores had improved significantly 1 year post-chemotherapy (T0 vs. T2: *p* = 0.001), with further improvements at T3 and T4 (T0 vs. T3: *p* = 0.014; T0 vs. T4: *p* = 0.002) (Figure 1, Table 3). However, there was an increase in the prevalence of erectile dysfunction at T5, with a significant reduction in ED domain scores; this seemed to persist at T6. Compared to controls, ED scores remained significantly worse at T1 then returned to a level comparable with healthy controls (Table 3).
- OF domain scores showed a trend of improvement from the baseline (*p* = 0.070), but pairwise comparisons against both baseline and controls did not reach statistical significance (Table 3).
- SD, IS, and GS domain scores, although showing a trend of improvement, did not differ significantly from the baseline at any time points; however TC patients scored significantly worse than the controls for the entire duration of the study (Table 3).

**Table 3:** IIEF scores of testicular cancer and control group: continuous data are presented as mean ± SD, median (in brackets) and 25–75th percentile of data distribution, while categorical data as percentage and counts.

	**ED domain**	**OF domain**	**SD domain**	**IS domain**	**GS domain**	**Erectile dysfunction (%)**
T0	22.7 ± 9.1^a^ (27.0)	8.2 ± 1.9 (10.0)	7.5 ± 1.9^a^ (8.0)	8.3 ± 4.7^a^ (10.0)	7.4 ± 2.6^a^ (8.0)	37.8%^d^ (91/241)
241 pts	20.0–29.0	8.0–10.0	6.0–9.0	7.0–12.0	6.0–10.0	
T1	24.1 ± 8.6^c^ (28.0)	8.8 ± 2.4 (10.0)	8.0 ± 1.7^b^ (8.0)	8.7 ± 4.2^b^ (10.0)	7.9 ± 2.3^a^ (8.0)	28.4%^d^ (21/74)
74 pts	24.0–30.0	9.0–10.0	7.0–9.0	8.0–11.0	8.0–10.0	
T2	25.8 ± 7.1 (29.0)	8.8 ± 2.3 (10.0)	7.6 ± 1.7^b^ (8.0)	9.4 ± 3.6^b^ (10.0)	7.9 ± 2.2^a^ (8.0)	23.6%^d^ (26/110)
110 pts	26.0–30.0	9.0–10.0	7.0–9.0	8.0–12.0	7.0–10.0	
T3	26.5 ± 6.3 (29.0)	8.7 ± 2.3 (10.0)	7.6 ± 1.6^b^ (8.0)	9.7 ± 3.7^b^ (10.0)	8.0 ± 2.2^b^ (8.0)	18.3%^d^ (11/60)
60 pts	27.0–30.0	8.0–10.0	7.0–9.0	9.0–12.0	7.0–10.0	
T4	26.9 ± 5.7 (29.0)	9.2 ± 1.7 (10.0)	8.0 ± 1.5^b^ (8.0)	10.0 ± 3.3^b^ (11.0)	8.5 ± 1.7^b^ (9.0)	16.0%^d^ (12/75)
75 pts	27.0–30.0	9.0–10.0	7.0–9.0	9.0–12.0	8.0–10.0	
T5	24.9 ± 8.0 (28.0)	8.0 ± 2.9 (10.0)	7.5 ± 1.8^b^ (8.0)	8.9 ± 4.0^b^ (9.0)	7.8 ± 2.3^a^ (8.0)	25.4%^d^ (17/67)
67 pts	25.0–30.0	6.0–10.0	6.0–9.0	8.0–12.0	7.0–10.0	
T6	25.2 ± 7.3 (28.0)	9.0 ± 2.0 (10.0)	7.7 ± 1.6^b^ (8.0)	9.8 ± 3.6^b^ (10.0)	8.2 ± 2.0^c^ (8.0)	30.5%^d^ (11/36)
36 pts	24.0–30.0	8.0–10.0	7.0–9.0	9.0–12.0	8.0–10.0	
CTR	27.9 ± 2.6 (28.5)	8.9 ± 1.2 (10.0)	8.9 ± 1.2 (9.0)	12.6 ± 1.9 (13.0)	9.0 ± 1.3 (9.0)	9.9% (22/223)
223 pts	27.0–30.0	8.0–10.0	8.0–10.0	11.5–14.0	8.0–10.0	
*P*-value	<0.001	0.068	<0.001	<0.001	<0.001	//

*(Kruskal Wallis test with Bonferroni correction for multiple comparisons). EF, erectile function; OF, orgasmic function; SD, sexual desire; IS, intercourse satisfaction; GS, general satisfaction*.

a p < 0.001 vs. Controls

b p < 0.01 vs. Controls

c p < 0.05 vs. Controls

d *χ^2^ p < 0.001 vs. Controls*.

### Sex Hormone Analysis

The prevalence of biochemical hypogonadism (total testosterone <8.0 nmol/l) was 4.1% in the TC group; there were no hypogonadal patients in the control group. The Kruskal Wallis test with *post-hoc* corrections for multiple comparisons (Bonferroni) revealed that gonadotropin (both FSH and LH) levels in the TC patients were higher both pre-chemotherapy and at T1 and T2 than in the controls (all *p* < 0.001). Total testosterone at T0 was significantly lower than in the controls (*p* < 0.001), but there was no difference from the control groups at T1 or T2 (Table 4). Finally, no significant correlation was found between total testosterone levels and the score of any IIEF-15 domain.

**Table 4:** FSH, LH and total testosterone of testicular cancer and Control group: continuous data are presented as mean ± SD, median (in brackets) and 25–75th percentile of data distribution, while categorical data as percentage and counts.

	**FSH (mUI/ml)**	**LH (mUI/ml)**	**Total testosterone (nmol/l)**	**Biochemical hypogonadism (%)**
T0	7.6 ± 6.3^a^ (6.1)	4.6 ± 4.1^a^ (4.0)	17.9 ± 6.1^a^ (17.0)	4.1% (8/194)
194 pts	4.0–9.8	2.7–5.6	13.3–20.9	
T1	14.3 ± 8.9^a^ (12.3)	6.6 ± 3.5^a^ (5.6)	19.2 ± 6.7 (19.0)	4.4% (3/68)
68 pts	7.3–19.7	3.7–8.2	15.0–21.6	
T2	13.0 ± 8.5^a^ (10.0)	6.8 ± 6.1^a^ (5.3)	17.9 ± 5.7 (17.9)	2.8% (2/71)
71 pts	7.1–18.5	3.8–7.4	13.5–20.5	
CTR	4.7 ± 4.5 (3.5)	3.7 ± 1.8 (3.3)	20.2 ± 7.1 (19.4)	0.0% (0/223)
223 pts	2.3–5.4	2.4–4.6	15.1–24.4	
*P*-value	<0.001	<0.001	0.002	//

*(Kruskal Wallis test with Bonferroni correction for multiple comparisons)*.

a *p < 0.001 vs. CTR*.

## Discussion

**Figure 2:**
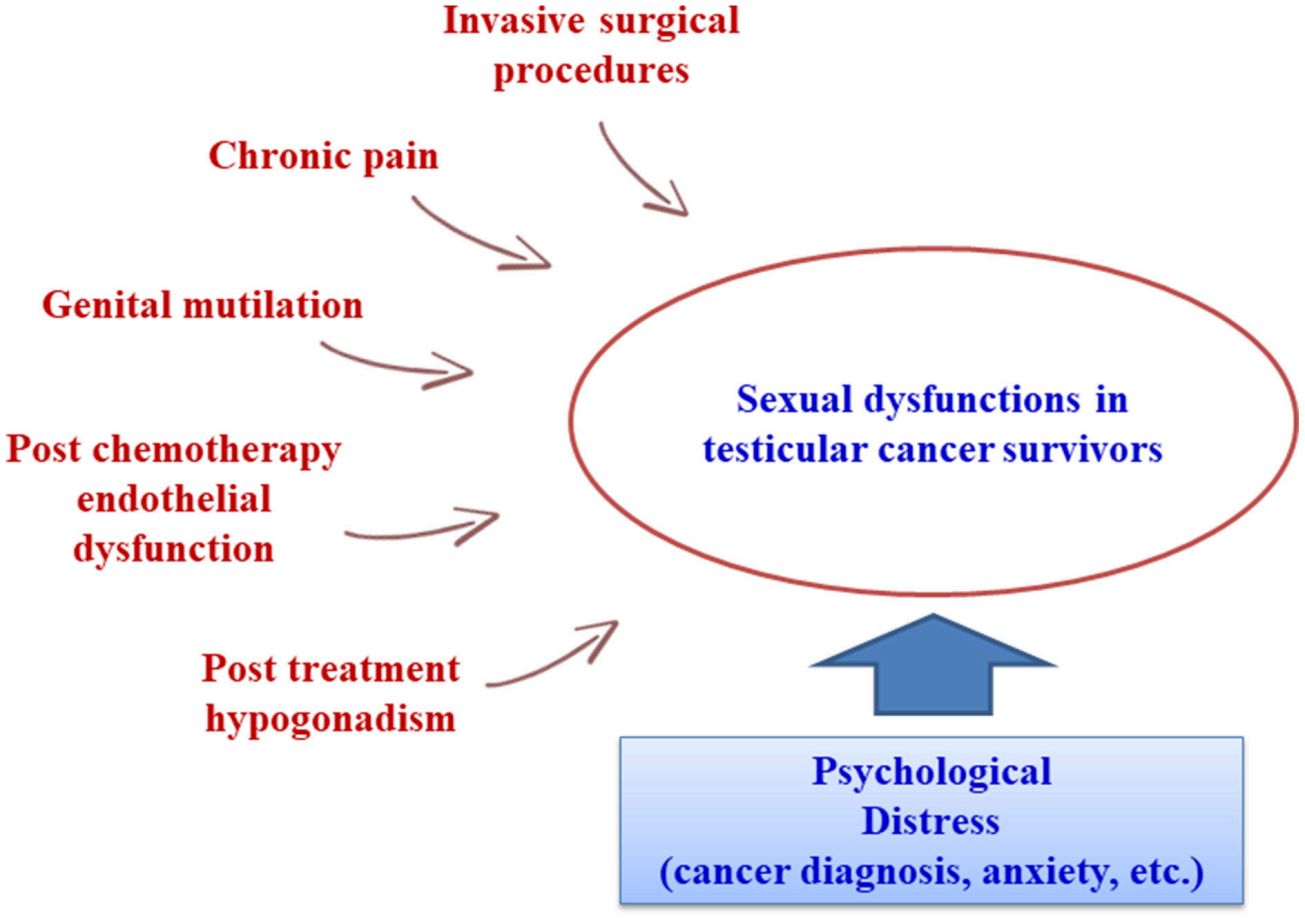
Determinants of sexual dysfunction in testicular cancer survivors.

The trend of reduced mortality for various cancers, including testicular cancer, has increased clinicians' awareness of the importance of long-term quality of life after surgery and chemo- and radiotherapy. Recent literature has focused on cancers involving the testes and genitalia (8, 12, 14, 15), but sexual function in male survivors of other frequent cancers has also been investigated (16, 17). In general, while TC survivors maintain or recover a good quality of life, investigation of their sex life reveals marked changes (18–20). Carpentier et al. highlighted that the diagnosis and therapy stages are associated with peak levels of anxiety and concern, which then drop in the post-treatment period. Similarly, stress-related central inhibition of sexual function results in a rise in libido, erection, and ejaculation disorders during cancer treatments (4). In fact, sexual dysfunctions in TC patients may arise from a combination of treatment-related physical side effects (genital mutilation, reduced testosterone levels, chronic pain, and other residual side effects) and psychological vulnerability (anxiety, fear, mood disorders, etc.) (Figure 2) (21). A possible underlying cause may be the induction of iatrogenic hypogonadism: in fact, orchiectomy, chemotherapy, and radiotherapy may all induce gonadal dysfunction. Our group recently found that a cohort of orchiectomized TC patients prior to cancer treatment had increased levels of gonadotropins and reduced testosterone in comparison with healthy controls, albeit still within the normal range (22). Some authors have shown that gonadotropin alterations persist after chemotherapy, while about 10% of patients may suffer from low total testosterone levels after treatment or have a higher risk of late onset hypogonadism (20, 23–26). However, other authors found that chemotherapy had only mild effects on hormone levels (27, 28). In contrast, radiotherapy may affect testosterone levels for up to 5 years in TC patients who received testicular irradiation for contralateral carcinoma *in situ* (29), but direct testicular irradiation is not a standard treatment for TC patients and current radiotherapy protocols probably have only a minor effect on testicular function (20). In any case, whether or not altered gonadotropin and testosterone levels are the only determinant of sexual dysfunctions in TC patients is still under debate. The already cited study by Huddart et al. found that about 10% of post-therapy TC patients had biochemical hypogonadism, with worse sexual function than observed in non-hypogonadal TC survivors (20). However, a later study by Lackner et al. (30) found a higher percentage of post-treatment hypogonadism (26%). These authors could not identify an unambiguous threshold level for testosterone associated with the onset of sexological symptoms, and hypothesized that each patient might have an individual threshold (30). In 2009, Eberard et al. published a caseload of 129 TC survivors 3–5 years post-therapy compared to an age-matched group of men without cancer, observing that TC survivors had a higher likelihood of low sexual desire (OR 6.7) and erectile dysfunction (OR 3.8) compared to controls, but that these conditions could not be predicted from the presence of hypogonadism (31). However, these results are limited by the lack of the pre-treatment status of the TC patients. Subsequent studies also failed to find a clear association between sexual dysfunctions and biochemical hypogonadism (28, 32–34). In conclusion, most of the literature evidence suggests that the high prevalence of sexual dysfunctions cannot be justified by the relatively low prevalence of biochemically detected hypogonadism. Another hypothesis could link sexual dysfunctions to specific treatment modalities. The side effects of several chemotherapy drugs include endothelial damage, angiopathy, and peripheral neuropathy, which may be linked to erectile and ejaculatory disorders (35, 36). Radiotherapy can cause sexual dysfunctions by inducing damage to the cavernous nerve and/or progressive fibrosis of the cavernous tissue and endothelial damage, which can become clinically evident through the onset of erectile dysfunction even several years post-treatment (37). However, the literature data are inconsistent, as several studies report no significant associations between sexual dysfunctions and specific treatment modalities (31), while others report the significant influence of either chemo- or radiotherapy (14, 15, 32, 38). The reason for this variability could be the use of different combinations of these therapies with different surgical procedures (tailored to the patient in relation to various clinical parameters such as stage, etc.), while individual variability might also induce different outcomes. Kim et al. reported that surgery combined with chemotherapy produced a higher incidence of reduced libido and ejaculatory disorders, while surgery combined with radiotherapy was followed by a greater incidence of erectile dysfunction (32). More recently, Bandak et al. observed that each treatment modality carried an increased risk of erectile and orgasmic dysfunctions, with multimodal treatment associated with the highest risk (15). Invasive surgical procedures such as retroperitoneal lymph node dissection are known to have a strong impact on sexual function (especially ejaculatory and orgasm disorders and impaired satisfaction) (12). Several studies have confirmed a worse sexological outcome after retroperitoneal surgery (lymph node dissection and/or re-surgery for relapse after chemotherapy) as a consequence of ejaculatory nerve damage during the procedure (10, 12, 21, 39, 40). Another issue is the trend of sexual dysfunction over time in TC survivors, as most literature studies are cross-sectional and only a few longitudinal studies are available. We currently expect a higher incidence of SD soon after the orchiectomy and the end of cancer treatment. Tuinman et al. found low IIEF scores post-orchiectomy and 3 months post-treatment, with significant improvements after 1 year of follow-up (41). These results were comparable to those of other studies focusing on SD within the first year after treatment (5, 42, 43). Long-term pre- and post-therapy comparisons are rare. Aass et al. found that sexual problems persisted in about 30% of TC survivors 36 months post-treatment (39), while Böhlen et al. reported no significant pre- vs. post-therapy differences in sexual function after at least 32 months of follow-up (44). Despite the general agreement on the presence of sexual dysfunctions in TC survivors, the absence of longitudinal long-term follow-up and the lack of standardization in the measurement of sexual function limit the assessment of their true patient burden. Data comparison and generalization are difficult, as different papers use a variety of tools and methods to evaluate sexual function. Moreover, in common with most sexological questionnaires, IIEF is poor at discriminating to what extent sexual dysfunctions are secondary to the organic sequalae of cancer treatments (45). In accordance with most of the available literature data, our results clearly show that TC patients undergoing orchiectomy and chemotherapy suffered from a higher degree of sexual dysfunctions than a control population of cancer-free subjects. These mainly presented as erectile dysfunctions, but also as impaired sexual desire and satisfaction. The incidence of orgasmic dysfunction did not differ significantly from controls. It is worth noting that the presence of these sexual dysfunctions at the baseline suggests that they might be induced by orchiectomy. However, it is difficult to find a biological relationship. We found a lower incidence (about 4%) of biochemical hypogonadism at the baseline (total testosterone < 8.0 nmol/l) than in other reports, but like them, we found no significant correlation with sexual function domains (20, 30, 31). This suggests that sexual dysfunctions are not explained by abnormal hormone levels consequent to orchiectomy and might instead be more closely associated with a psychological burden in these patients, which might coexist and be synergistic with treatment-induced hypogonadism. As the testicles are associated with masculinity, orchiectomy might well induce changes in body perception. In a caseload of 407 TC patients, Rossen et al. observed that about 17% had a reduced perception of masculinity induced by orchiectomy. This was associated with a 9-fold increased risk of erectile dysfunction and a 15-fold increased risk of sexual discomfort (10). Wortel et al. reported that after orchiectomy, up to 50% of patients might complain of a distorted perception of body image (42). All of our patients underwent the insertion of a testicular prosthesis. This may have positively influenced their body perception, as suggested in a study by Catanzariti et al. (43), but whether and to what extent this might have contributed to the improved IIEF-15 scores in our study is unknown, and should be investigated in further studies. We also detected a significant improvement in erectile function post-therapy. Although some degree of erectile dysfunction persisted at all time points, the incidence constantly dropped in the first 2 years after treatment (up to T4) and the IIEF-15 erectile dysfunction scores improved and were comparable to the controls 1 year after treatment (T2). There was also a trend of improvement in sexual desire and in both intercourse and general satisfaction, but follow-up showed that they remained significantly worse than in the controls. To our knowledge, the present study is the longest monocentric follow-up currently available for the sexological evaluation of TC patients. Unfortunately, the generalizability of data comparisons against healthy controls might be reduced for long term follow-up, as the increased percentage of patients with erectile dysfunction at T5 and T6 may simply be due to their increased age and the consequent possible onset of other factors (hypertension and other cardiovascular diseases, use of medications, etc.) increasing the incidence of sexual dysfunctions, independently of TC and its treatments.

In conclusion, our data, from a large caseload compared to a control group of similar age and strengthened by the use of a validated psychometric tool, indicate that TC patients need adequate sexological counseling following diagnosis/orchiectomy and prior to chemotherapy. Discussing these aspects with patients could help them to cope with the disease and to understand that their erectile function should improve within a year after the end of treatment. Future studies should identify subjects who are more likely to suffer from SDs, thus permitting the better follow-up of these patients and enabling them to be offered all the support they need to maintain a satisfactory sex life and, consequently, a good general quality of life.

## Data Availability

The datasets for this manuscript are not publicly available. If required, the data are available in our database upon request. Requests to access the datasets should be directed to donatella.paoli@uniroma1.it.

## Author Contributions

FL, FP and DP conceived the study; FP and FL wrote the article; FP, AP and FC acquired and analyzed the data; FL, FP and DP contributed to data interpretation; AR and AL manuscript revision.

### Conflict of Interest Statement

The authors declare that the research was conducted in the absence of any commercial or financial relationships that could be construed as a potential conflict of interest.
